# Electroconvulsive therapy portrayal in contemporary video games

**DOI:** 10.3389/fpsyt.2023.1336044

**Published:** 2024-01-05

**Authors:** Jozef Buday, Miroslav Neumann, Jana Žaludová Heidingerová, Tadeáš Mareš, Eva Magyarová, Hong Thai Le, Daniel Divácký, Gabriela Jirečková, Jakub Albrecht, Lucie Kališová, Marek Pol, Jakub Mahrík, Patrik Buday, Martin Anders

**Affiliations:** ^1^Department of Psychiatry, Faculty of Medicine, Masaryk University and University Hospital Brno, Brno, Czechia; ^2^Department of Psychiatry, First Faculty of Medicine of Charles University and General University Hospital, Prague, Czechia; ^3^Grammar School Bilíkova, Bratislava, Slovakia; ^4^Department of Psychiatry, Faculty of Health Studies, Krajska zdravotni, a.s, Jan Evangelista Purkyne University and Masaryk Hospital, Usti nad Labem, Czechia; ^5^Faculty Hospital Královské Vinohrady, Prague, Czechia; ^6^Institute of Clinical and Experimental Medicine, Prague, Czechia; ^7^Tatra Defence Vehicle, Kopřivnice, Czechia

**Keywords:** electroconvulsive therapy (ECT), electroshock therapy, electroshock device, videogame, stigma

## Abstract

Electroconvulsive therapy (ECT) is an important treatment modality in psychiatry, considered to be the most effective option for pharmaco-resistant affective and psychotic disorders. Despite its great efficacy, it still remains a rather controversial method, which hinders its full potential. It is feasible to say that in part, this controversy is caused by a largely negative image of ECT displayed through media. The depiction of ECT in movies has been studied and well documented in the past. The aim of our study was to provide an overview of how ECT is represented in video games - a form of media where ECT representation has been overlooked in scientific literature so far. As with movies, most of these portrayals are negative, depicting ECT as an obsolete, aggressive or torturous treatment method.

## Introduction

Electroconvulsive therapy (ECT) is an important treatment modality in psychiatry. It is considered to be the most effective option for pharmaco-resistant affective and psychotic disorders (1). Over the last decades, the application of ECT undergone radical changes—in terms of technological improvement, procedure and dosing, which has led to a significant minimalization of its adverse effects (2). Nonetheless, in the eyes of the public, it still remains a rather controversial method, which hinders its full potential (3). Several studies have unfortunately also pointed out that the negative image of ECT is also extended to other medical professions and students as well (4). It is feasible to say that in part, this controversy is caused by a largely negative image of ECT displayed through media. The depiction of ECT in movies has been studied and well documented in the past (5). According to Sienaert (5), between 1948 and 2016, ECT was portrayed in 52 movies, 21 TV programs and 2 Sitcoms, usually depicted as a memory-erasing, damaging, obsolete or downright torturous method. This image reached millions of viewers and likely added to the stigma that is associated with ECT.

In 2022, our team conducted a study aimed at the depiction of mental illness in popular video games (6). Video games are an important popular type of new media—it is estimated that in 2023, there will be 3.07 billion gamers (7), thus they constitute an important source of information able to reach wide audiences. As far as we are aware, there are currently no studies available that mapped the portrayal of ECT in video games, as was done with movies and other media forms in the past.

The aim of our study was to provide an overview of how ECT is represented in video games—we believe that this knowledge may be important and informative for psychiatrists and ECT practitioners, since they should be aware of possible misconceptions relayed to audiences via video games, so that they are prepared to explain them to patients or their concerned relatives, if the option of ECT application ever arises. We would like to add that this article should not be perceived by any means as a review or critique of the presented video-games, but merely an observation as to how ECT treatment is portrayed.

## Methods

For the purposes of this research, we have used two separate strategies to identify video games that contain the portrayal of ECT in order to cover as much ground as possible. We have essentially combined the approaches of our previous study (6) and that of Shapiro and Rotter (8) and Ferrari et al. (9).

First, we used the Google search engine and Steam (PC gaming platform) to research all combinations of the following terms: “Electroconvulsive therapy,” “ECT,” “Electroshock therapy,” “Shock therapy,” “Electroshock treatment,” “Electric therapy,” with “Video Games,” “PC games,” “Games.” On purpose, we also used the older and more lament terms of “Electroshock therapy” and “Shock therapy” which are more widely known among the public. In this search, we reviewed the first 200 search results and if a text or a video-link confirming the presence of such a portrayal was identified, we would copy this to our Excel electronic database.

Second, similar to our previous article, we used the statistical data of the best-selling games in the United Kingdom—UK Games Charts (10), that are released and made publicly available by a trade association for the UK’s games and interactive entertainment industry—Ukie. This database contains the top 100 selling games since 1999 to 2019. We used the Google search engine to combine every game title from this list with the terms “Electroconvulsive therapy,” “ECT,” “Electroshock therapy,” “Shock therapy,” “Electroshock treatment,” and “Electric therapy.” Since other years were not available on Ukie, for 2020–2022, we used the data of top selling games from the Interactive Software Federation of Europe and cross referenced them with the terms mentioned above (11). We have reviewed the first 100 search results with each game title and if a text or a video-link confirming the presence of an ECT portrayal was found, we would copy this to our Excel electronic database (either a descriptive text or link to a video footage). Some games remain best-sellers for multiple years, and thus after removing duplicate titles, we ended up reviewing 528 titles in total.

The extracted data was thoroughly read (or viewed if it was a video link) using a general inductive approach (12) and manual coding. We reduced overlap, redundancy and results where some keywords appeared out of context. We have also excluded indirect use of the term “Electroshock therapy” that could be associated with ECT, but does not portray the medical procedure itself (for example, in *Dungeon Siege II*, there is an achievement titled “Electroshock therapy” that the player receives for electrocuting 10 enemies with an electricity based weapon). We also further analyzed whether these portrayals were positive, neutral or negative and whether the portrayal of ECT was realistic or not when it comes to the procedure itself. Negative portrayal was assigned when a game showed or mentioned that ECT had clearly detrimental effects on its recipients or when it was practiced as a method of torture. Neutral representation was assigned when ECT was mentioned or portrayed in the game without the audience knowing what sort of effect the treatment had on the recipient. And finally, a positive representation would be assigned if ECT had a positive effect on the recipient. The data was subsequently organized by game title and summarized in Table 1.

**Table 1:** Games that depict or mention electroconvulsive therapy (ECT) explanation to some words in the table.

Game title	Developer and country	Genre	Context	Impact of ECT on subject	Direct portrayal?	Length of portrayal	Positive/Neutral/Negative
AC Creed: Syndicate (2015)	Ubisoft (Canada)	Action-Adventure	ECT is portrayed as a method used to brainwash subjects who receive it during a quest in a mental asylum	Subjects receiving ECT are “zombified”	Yes	A quest completed in approx. 30 min	Negative
Batman: Arkham series (2009–2015)	Rocksteady Studios (UK)	Action-Adventure	It is mentioned and implied that several characters were treated with ECT as a method of torture in a mental asylum	ECT affected the characters by changing their personalities and memories	No	Mentioned in the game lore/documentation	Negative
Bioshock (2007)	2 K (USA)	First person shooter with horror elements	Mentioned in the lore that one of the characters was subjected to ECT as a method of torture	Unknown	No	Mentioned in the game lore/documentation	Negative
Bioshock: Infinite (2013)	2 K (USA)	First person shooter with horror elements	Mentioned in the lore that one of the characters was subjected to ECT as a method of torture	Unknown	No	Mentioned in the game lore/documentation	Negative
Blackstone Chronicles (1999)	Legend Entertainment (USA)	Adventure with horror elements	While exploring a mental asylum, the player stumbles across an ECT device which the main character comments as being used to brainwash patients	Implied by the main character that it was being used as punishment	No	Comment by the main character during the game	Negative
Breakout 13 (2022)	Alt Lab (China)	Adventure	Patients in a “correctional camp” receive ECT as a part of punishment to rectify “Internet addiction”	Used as a punishment for main characters, inducing behavior change and memory alteration	Yes	We witness several ECT sessions from the point of view of the subject	Negative
Cyberpunk 2077 (2020)	CD Projekt Red (Poland)	Role-playing game	Mentioned being used as torture in a corrupted mental asylum for an honest policewoman during one of the quests in the game	Used as torture for a side character during a quest in the game	No	Mentioned in the game lore/documentation	Negative
Dead by Daylight (2016)	Behaviour Interactive (Canada)	Survival horror	One of the villains is inspired by dr. Yang Yongxin, who used ECT in China to treat “Internet Addiction,” the character is playable and can use electrical energy to kill other players	The character can use “electro-convulsive” power to stun/kill other players in the game	No	Playable character associated inspired by scandalous real life events	Negative
Mysteries of the Fence (2017)	Orange Light Game (China)	Adventure	Patients in a “correctional camp” receive ECT as a part of punishment to rectify “Internet addiction”	Used as a punishment for main characters, inducing behavior change and memory alteration	Yes	We witness several ECT sessions from the point of view of the subject	Negative
Outlast (2013)	Red Barrels (Canada)	Survival horror	The player explores a mental asylum, ECT is mentioned in the game documentation with a negative connotation, to calm down patients	Used as a mean to alter behavior in patients	No	Mentioned in the game lore/documentation	Negative
Silent Hill Homecoming (2008)	Team Silent (Japan)	Survival horror	ECT is mentioned as being used on one of the characters in the game documentation	Used as a mean to treat depression, the effect is unknown	No	Mentioned in the game lore/documentation	Neutral
The Evil Within (2014)	Tango Gameworks (Japan)	Survival horror	A device used to deliver electrical shocks via a helmet is used by the main character to upgrade his skills, the game takes place in a haunted mental asylum	Used as a means to upgrade the skills of the main character	No	Each time the player wishes to progress his skills	Neutral
The Town of Light (2016)	LKA (UK)	Adventure	The main character receives ECT as a part of their treatment in a mental asylum (however, implied use as punishment), technically, the procedure is depicted realistically	Used to alter the behavior and memories of the main character	Yes	A direct depiction of an ECT applied to a main character lasting several minutes	Negative

Lore: is the background information that enriches the game’s story, characters, and setting. Quest: is a task in video games that a player-controlled character, party, or group of characters may complete in order to gain a reward. Documentation: various letters and documents that the player can read and interact with in the game.

Games that were found to contain portrayal or mention of ECT were played by members of our team to directly confirm its presence, besides that, we also used YouTube footage for this confirmation, however, this was not always possible to find, especially with older titles. Video game titles were played on a personal computer (PC).

Explanation of some words used in the methods for readers that are not familiar with videogames are below.

Lore: is the background information that enriches the game’s story, characters, and setting.

Quest: is a task in video games that a player-controlled character, party, or group of characters may complete in order to gain a reward.

Documentation: various letters and documents that the player can read and interact with in the game.

Achievement: a digital reward that signifies a player’s mastery of a specific task or challenge within a video game, usually acknowledged by a text pop-up once it is completed.

## Results

We have found a total of 13 representations of the portrayal of ECT or direct reference to this treatment in video games. Most of these representations are negative and associated with video games from the horror genre. Out of these 13 representations, we have identified 4 in which ECT is directly portrayed in a graphic manner. In the other games ECT is mentioned in the lore or various documentation.

There was only one representation (*The Town of Light*) that can be considered to be a realistic depiction of unmodified ECT (that is, ECT without the use of anesthesia and muscle relaxation, which was used mainly during the 1930s and 1940s). *Blackstone chronicles (1999)* is a visual novel game that included a picture of an ECT device with correct basic information, however, the main character internally talked about the method in stigmatizing ways.

Unfortunately, we have not been able to identify a single video game that portrays ECT in a positive way.

Most notable depictions of ECT In video games include:

### The town of light (2016)

In this psychological horror game, the player is put in the role of a female character Renée, who is arguably suffering from Post-Traumatic Stress Disorder (PTSD). The game takes place in a former Psychiatric Hospital “Ospedale Psichiatrico di Volterra,” in Italy, which is currently abandoned, but the main character decided to visit it again in order to relive her hospitalization there, which took place in the 1940s. Renée is reliving this experience via traumatizing flashbacks, during which it is apparent that she was a victim of sexual abuse, torture and malpractice. She fell in love with one of her co-patients, however, as a punishment during her stay, they were separated and she was convinced by the staff that this person was just a figment of her imagination and that she allegedly suffered from a psychotic illness, which turns out to be untrue during the game. One of these flashbacks depicts the usage of unmodified ECT which was used as a punitive measure. It is interesting to see that the technical portrayal of the method is actually quite precise in this video game. We can see that there are several nurses around her bed (presumably to hold her down during the convulsive phase so that an injury is avoided), a doctor who is manipulating parameters on a realistic historical ECT device and a nurse who puts electrodes in a bitemporal arrangement, still commonly used today. It is implied in the game that the method was used to wipe the memory of the main character.

*Mysteries of Fence (2017) and Breakout 13 (2022)* are video games that are unfortunately inspired by real life events. In 2008, there have been numerous reports on the treatment of “internet addiction” in the Shandong province in China with ECT (13). This was eventually picked up and confirmed by the Chinese Ministry of Health, the incident ended with a ban on the usage of ECT in China for this “indication” (14). Both games are adventures inspired by these events and follow a group of students who are tortured with ECT. The method is portrayed with the students being tied to chairs with a helmet placed on their heads, via which the psychiatrist would apply electrical shocks to the recipients—without anesthesia or muscle relaxation. The administration of ECT is preceded by an interrogation and it is clear that the method is used as a punishment for perceived undesirable behavior.

### Assassin’s creed: Syndicate (2015)

In this action-adventure game with stealth elements, the player is reliving the memories of twin assassins Jacob and Evie Frye. During the quest “Overdose,” the player is tasked to kill dr. Elliotson, a former surgeon who became obsessed with phrenology and is now working in an mental asylum, where he performs unethical experiments on human patients. One of these experiments is the usage of ECT. The device is depicted as a large, Victorian steam-like engine that produces electrical current, that is applied to the head of a patient, who is clearly in a lot of pain during the application. The player is literally tasked to “stop the electroconvulsive therapy session,” which is performed by two guards without any medical background, who are not even aware why the patient is receiving this form of treatment. It is implied that the method was used to “zombifie” its recipients.

## Discussion

Similar to movies, ECT is largely portrayed in a negative light in video games and is usually connected to the horror genre. Most games represent ECT as a cruel, barbaric or otherwise torturous method associated with very asylum-like settings. However, it seems that the representations of ECT in videogames is not as frequent as in cinema—Sienaert (5) for instance have found 82 instances in total of ECT depiction in movies and TV shows, compared to our 13, of which only 4 contain direct graphic representation of ECT. Movies and TV shows are usually much shorter than video games and the scenes were ECT is represented take a larger portion of the narrative in total, which might make them more memorable. In video games however, these depictions are only a fraction of the total gameplay time—for example, an average player would take 6 h to complete the Town of Light, and 19–32 h to complete Assassin’s Creed: Syndicate. The scenes where ECT is represented in video games are very short and the player spends a majority of the time doing unrealistic and fantastical things within the gameplay, which does not make ECT “stand out” as much in the grander picture. Most video games where ECT is represented are unrealistic and associated with supernatural and horror elements.

Currently, there is no strong evidence that playing video games influences aggression, impulsivity-related constructs, mood, anxiety, empathy, interpersonal competencies or executive control functions (15). While there have been a limited number of studies that showed a correlation between violent video games and short-term aggression, these studies are not consistent and difficult to replicate (15). Similarly, we also think that ECT depiction in videogames and its ability to influence the opinions of their players is limited. However, ECT practitioners should be aware that these depictions in modern video games exist and might contribute to a negative opinion about ECT prior to receiving information about the real method. For instance, the depiction of unmodified ECT in *Town of Light* is quite realistic, yet, a player without deeper knowledge about this topic might come to the conclusion that ECT is still performed without anesthesia or has catastrophic adverse effects. Although the technical aspect of the depicted ECT in The *Town of Light* is quite accurate, the player does not receive any additional information about the method and thus might come to the conclusion that it is obsolete or was only used in the past in order to torture or modify behavior of patients. We think it might be prudent for future game developers, if they choose to include a direct depiction of this medical procedure, to contain more visible disclaimers about the depiction being used only for narrative purposes and perhaps offer a link to information about the real-life procedure.

It is important to state here that there were real-life incidents of ECT malpractice, some not that far in the past, such as the scandalous usage of ECT in a group of young students allegedly suffering from “video games addiction” in China (14). It is important to note that nowadays, ECT is applied under a brief general anesthesia in developed countries, has specific indications (pharmaco-resistant affective and psychotic disorders) and in principle requires consent from the patient (16–19). The application of ECT without the patient’s consent is not common and constitutes life-threating conditions, such as syndrome of catatonia or severe psychotic illnesses with malnourishment (20). Although adverse effects after ECT do exist, as with any other treatment modality in medicine, the cognitive adverse effects were drastically minimized compared to insulin shock therapies or unmodified ECT procedures that were used in the past (21). In fact, the time to lucidity in right-unilateral ECT application is on average 12 min, which allows the application of ECT in out-patient settings, which is in stark contrast with the general representation of ECT in video games as causing drastic cognitive damage/or require hospitalization in an asylum (22). Future studies might also want to compare other treatment modalities represented in video games—such as the use of pharmacological injections or psychotherapy, to analyze whether they are also represented in such a negative light.

To this date, ECT remains unparalleled when it comes to treatment efficacy and there is a general consensus, based on the result of the STAR*D study (23), that it should be offered to pharmaco-resistant patients sooner, rather than later (1). This is especially important to remind, since ECT is stigmatized not only among the general population, but medical professionals as well.

## Study limitations

We have used the Google search engine based in Czech Republic to conduct our research—we do not exclude the possibility that a different location might produce different results. Similarly, for the second part of our research, we have mostly used UK Game Charts, which represent the most sold games in the United Kingdom, thus it is possible that due to cultural differences, the best-selling games in other regions of the world might be different. We have also limited our search to PC and console games and did not conduct a search on other platforms, such as mobile games.

We have decided to exclude videogames that mentioned the word “electroconvulsive therapy” but did not portray/reference the direct medical procedure, however, we should add that even these mentions might contribute to the overall stigmatization of ECT (as the example in *Dungeon Siege II* mentioned above).

We have also not used separate raters for the individual depiction of ECT and whether they perceived this depiction as positive/neutral/negative and assessed this as a group, therefore we cannot provide the readers with an intraclass correlation coefficient.

Finally, although we have conducted a search on both Steam and the Google search engine, it is possible that certain indie games that are not popular and do not reach a wider audience also eluded our list.

## Conclusion

The portrayal of ECT in video games is not common. Since 1998, we have only identified 13 games that contain a clear depiction of ECT or a direct reference to this treatment modality. The majority of these portrayals take place in games with horror like elements. As with movies, most of these portrayals are negative, depicting ECT as an obsolete, aggressive or torturous treatment method. Most representations of ECT are only a small fraction of content in a wide context of unrealistic features within video-games and further research is needed to determine to what extent these portrayals shape the opinion of the public. Due to ECT being a method that is still stigmatized both among general and medical public, we find it important to relay information as to how it is portrayed in new media.

## Data availability statement

The original contributions presented in the study are included in the article/supplementary material, further inquiries can be directed to the corresponding author.

## Author contributions

JB: Conceptualization, Formal analysis, Methodology, Project administration, Writing – original draft, Writing – review & editing. MN: Data curation, Investigation, Methodology, Writing – original draft. JŽ: Formal analysis, Visualization, Writing – original draft. TM: Data curation, Investigation, Methodology, Writing – review & editing. EM: Data curation, Formal analysis, Writing – review & editing. HT: Data curation, Investigation, Writing – review & editing. DD: Data curation, Formal analysis, Investigation, Methodology, Writing – review & editing. GJ: Data curation, Methodology, Writing – review & editing. JA: Data curation, Methodology, Writing – review & editing. LK: Formal analysis, Project administration, Validation, Writing – review & editing. MP: Data curation, Investigation, Methodology, Writing – review & editing. JM: Data curation, Investigation, Methodology, Writing – review & editing. PB: Data curation, Investigation, Writing – review & editing. MA: Supervision, Writing – review & editing.
